# Diagnosing anemia: Challenges selecting methods, addressing underlying causes, and implementing actions at the public health level

**DOI:** 10.1111/nyas.14996

**Published:** 2023-04-15

**Authors:** Maria Nieves Garcia-Casal, Omar Dary, Maria Elena Jefferds, Sant-Rayn Pasricha

**Affiliations:** 1 Department of Nutrition and Food Safety, World Health Organization, Geneva, Switzerland; 2 Bureau for Global Health, US Agency for International Development, Washington, DC, USA; 3 Division of Nutrition, Physical Activity and Obesity, Centers for Disease Control and Prevention, Atlanta, Georgia, USA; 4 Population Health and Immunity Division, The Walter and Eliza Hall Institute of Medical Research, Melbourne, Victoria, Australia

**Keywords:** anemia, diagnosis, equipment, methods, samples

## Abstract

Accurate and affordable tools for diagnosing anemia and its main determinants are essential for understanding the magnitude and distribution of the problem and the appropriate interventions needed for its timely prevention and treatment. The objective of this review is to address methods, equipment, and sample-related and quality control aspects of hemoglobin measurement for anemia diagnosis. Also, other iron-, infectious-, and genetic-related causes of anemia are addressed in individuals and populations. The best practice for hemoglobin determination is the use of venous blood, analyzed on automated hematology analyzers, with high-quality control measures in place. The importance of a correct anemia diagnosis is highlighted by the cost of a misdiagnosis. A false-negative diagnosis may result in missing out and not treating anemia, its causes, and its adverse effects. On the other hand, a false-positive diagnosis may result in the provision of unneeded treatment or referral for expensive laboratory tests to determine a cause of anemia, wasting valuable resources and risking causing harm. At the individual level, clinicians must understand the causes of absolute and functional anemia to diagnose and treat anemia at the clinical level. Actions toward anemia diagnosis and control at public health levels require global, regional, and country actions that should cover general and context-specific characteristics.

## INTRODUCTION

This paper was developed as an input paper for the *Comprehensive framework for integrated action on the prevention, diagnosis and management of anemia* led by the World Health Organization (WHO). This is one of five papers that will serve as working papers providing insights to help diagnose anemia and its causes with acceptable accuracy and precision in individuals and populations, and to prioritize and maintain actions to accelerate reductions in the prevalence of anemia. The four additional papers address the objectives and purpose of the *Comprehensive framework for integrated action on the prevention, diagnosis and management of anemia* and the topics of determinants of anemia, preventive and therapeutic interventions, and the integrated management of anemia across the life course.^1-4^

The objective of the paper is to review methods, equipment, and sample-related and quality control aspects of hemoglobin (Hb) measurement for anemia diagnosis. Also, since Hb measurement cannot alone determine the cause of anemia, other iron-, infectious-, and genetic-related causes are addressed, as well as major gaps or challenges in generating effective action and impact in anemia prevention and control.

## DEFINITION AND CLASSIFICATION OF ANEMIA

Anemia is a condition in which the number of red blood cells (RBCs) or the Hb concentration within them is lower than normal.^5^ Anemia may produce clinical symptoms, such as fatigue, palpitations, headache, and shortness of breath, and signs, such as conjunctival and palmar pallor, which in spite of having low sensitivity and moderate specificity to diagnose anemia,^6^ are still useful when laboratory assessment is limited.^7,8^ In clinical practice and in public health, the measurement of Hb concentration is the most common indicator and assessment method used to define anemia.

Hb concentration varies by age, sex, pregnancy and health status, and genetic and environmental factors.^9,10^ In the newborn, normal Hb concentrations are between 170 and 210 g/L, decreasing during the first months of life to a value around 100 g/L for 6- to 9-month-old infants,^11^ before increasing again in childhood to approximately 110 g/L up to 59 months, to 115 g/L up to 11 years and to 120 g/L for adult females and 130 g/L for adult males.^12-15^

Sex differences in Hb concentrations begin in puberty (because of the negative effect of menstruation on iron stores in women and the positive effect of testosterone on erythropoiesis in men) and continue throughout the reproductive years.^16,17^ During pregnancy, because of the expansion of blood volume and consequent dilution effect, Hb concentration declines during the first and second trimesters, rising gradually again late in the third trimester.^4,18-20^

Elevation above sea level of the place of residency and smoking are known to increase Hb concentrations as a physiological response to hypoxia, as more Hb is necessary to compensate for the reduction in oxygen saturation, triggered by an increase in erythropoietin production. WHO currently has recommendations for adjustments of Hb concentration by those two factors, which can be used to improve the accuracy of the estimated prevalence of anemia among those populations.

It has been suggested that race is an additional biological factor that determines Hb distribution and, consequently, the prevalence of anemia,^21-24^ while other studies do not support the use of race as a biological factor because it is a social construct and suggest that inequitable social determinants of health may contribute to these differences.^25,26^ There is some evidence from genetic studies among European populations that several genetic variants are associated with alterations in iron status, indicating that genetic ancestry may contribute to variations in Hb concentrations and anemia prevalence globally.^22^ However, genetic ancestry is related to but not equivalent with race, which is a social construct.^27^ This has led some to call for the removal of race-based guidance in hematology because clinical decisions based on phenotypic or patient-identified race may contribute to existing racial health disparities.^28^ WHO documents report that black individuals have lower Hb concentrations than white individuals (approximately by 10 g/L); these differences are not explained by health, nutrition, or socioeconomic status, and are observed across the age spectrum.^10^ However, other WHO documents do not currently consider race or genetic ancestry.^29^

Anemia can be classified by underlying cause, for example, increased red cell loss compared with reduced production. Anemia may be microcytic (e.g., due to iron deficiency or thalassemia), normocytic (e.g., due to inflammation), or macrocytic (e.g., due to vitamin B12/folate deficiency, liver disease, myelodysplasia, or hypothyroidism).^10^

Usually, morphological characterization is more convenient to support the diagnosis and indirectly the treatment in connection with the laboratory results and clinical evaluation, although it is of limited use to determine the etiology of anemia.

Anemia can also be classified by severity at the individual level and for populations as a public health burden. In the two cases, anemia may be classified as mild, moderate, or severe. The current classification of anemia as a public health problem is shown in Table 1.

## HEMOGLOBIN CUTOFFS TO DEFINE ANEMIA

The current WHO cutoffs to define anemia at the individual level were established in 1968 after technical meetings of an expert group comprising clinical and public health specialists working with the evidence available at the time, which comprised data from five studies of predominantly Caucasian populations in Europe and North America.^29^ These cutoffs were slightly modified in 2001^30^ and are the current WHO cutoffs^29^ (Table 2).

WHO is updating guidelines for the use and interpretation of Hb concentrations to diagnose anemia at the individual and population levels.^31^ This project follows established WHO guideline development procedures and will culminate in an updated guideline for clinical and public health use. Any potentially revised cutoffs may be critical, not only for correctly identifying individuals with anemia but also for determining public health problems among populations, implementing interventions, or for evaluating performance and comparability of existing tests for Hb.

## CAUSES OF ANEMIA

WHO has described the determinants of anemia^10^ as biological (nutrient deficiencies and other forms of malnutrition, growth, physiological state, sex, age, and race); related to infection and inflammation (soil-transmitted helminth infections, schistosomiasis, malaria, HIV, tuberculosis, low-grade inflammation); genetic Hb disorders; blood loss/contraception; and social, behavioral, and environmental determinants.^2^

The most common micronutrient deficiency associated with anemia is iron deficiency. Iron deficiency can be absolute when body iron stores are insufficient to satisfy iron needs, and functional when despite sufficient iron stores the organism cannot utilize iron because both the mobilization and absorption of iron are reduced in order to limit access of iron to pathogenic organisms. Both types of iron deficiency may coexist in the same individuals or populations.^32^ Other less common micronutrient deficiencies that have been reported to cause or contribute to anemia include vitamins A, B2, B6, B9, B12, C, D, and E, copper, and zinc. Micronutrient deficiencies could occur when intake of micronutrients cannot meet the body’s demands over a period of time due to low consumption, low bioavailability, high content of inhibitors or low content of enhancers of absorption, increased needs (i.e., during periods of rapid growth like infancy and adolescence, or during pregnancy), and/or increased losses.^33^

Non-nutritional causes of anemia include inflammation caused by infection (e.g., tuberculosis, malaria, HIV) and noninfectious causes (e.g., cancer, organ failure, autoimmune disease); parasites causing bleeding (e.g., hookworm, schistosomiasis); and genetic conditions (e.g., thalassemia and sickle cell disease, glucose-6-phosphate dehydrogenase (G6PD) deficiency, red cell membrane disorders). Upstream risk factors include environmental or support factors (poor sanitation, unsafe drinking water, inadequate personal hygiene, economic and political gaps, low institutional capacity/resources, adverse climatic/environmental conditions).^10,34^ Other upstream factors associated with anemia, particularly in women, include poverty, obesity, low education level, poor household wealth, cultural norms, lack of empowerment, rural living, inadequate health care, low nutrition knowledge, inappropriate health policies, limited access to health care, inadequate maternal and childcare, and vulnerability of women and children (early onset of childbearing, high parity, and short birth spacing).^33^

## DIAGNOSING ANEMIA

Accurate, precise, acceptable, and affordable tools for diagnosing anemia and its main determinants^2^ are essential for understanding the magnitude and distribution of the problem and the appropriate interventions needed for prevention and treatment.^3,4^

Anemia is commonly diagnosed through the measurement of blood Hb concentration. However, anemia can also be assessed using hematocrit (packed cell volume), and with more causal specificity through RBC parameters, such as mean cell volume and mean cell Hb concentration, and reticulocyte count. Other indicators are blood film examination, Hb electrophoresis (or high-performance liquid chromatography), micronutrient biomarker measurement, Hb color scale, or clinical changes.^35,36^ Using different indicators will identify different individuals as having anemia as they measure different metabolites/processes.

### Measuring Hb

Hb measurement should ideally be performed in well-equipped clinical laboratories. Methods for determining Hb are mostly based on the spectrophotometric properties of Hb or its derivatives, such as cyanmethemoglobin (or any other Hb derivative), which is considered the gold standard for Hb estimation.^9,37,38^ Several other methods are available based on the color of Hb, such as the hemoglobin color scale, the Sahli method, the Lovibond–Drabkin technique, and the Tallqvist technique. Another method is based on the specific gravity of blood that is compared against that of a specific copper sulfate solution. Each method has a different principle and its own advantages and disadvantages. Some of the most commonly used methods for Hb determination are presented in Table 3. All of them, except for noninvasive methods, can be performed using arterial, venous, or capillary blood samples.

The cyanmethemoglobin method, the gold standard for Hb determination,^9,39^ is a relatively inexpensive and stable method with an internationally accepted reference standard calibrator, although the potassium cyanide is toxic at high concentrations, making the management of the safe disposal of this reagent difficult. In this method, blood is mixed with the Drabkin’s solution (a mix of potassium cyanide, potassium ferricyanide, and potassium dihydrogen phosphate). The erythrocytes are lysed and Hb is released. Potassium ferricyanide transforms Hb into methemoglobin that combines with potassium cyanide to produce hemiglobincyanide (cyanmethemoglobin). The absorbance at 540 nm is read in a spectrophotometer and compared with that of the standard hemiglobincyanide solution and the Hb concentration in the sample calculated. Most Hb derivatives (oxyhemoglobin, methemoglobin, and carboxyhemoglobin, but not sulfhemoglobin) are converted to hemiglobincyanide and, therefore, measured by this method.^39^

Automated hematology analyzers generally use the cyanmethemoglobin method or produce other Hb derivatives and determine the absorbance of the specific formed derivative against the appropriate standard.^40^ Some automated cell counters use sodium lauryl sulfate (SLS) instead of cyanide-containing reagents, although those commercially available solutions containing SLS are less stable than the Drabkin solution. Sodium lauryl sulfate converts Hb into methemoglobin in the order of oxyhemoglobin, hemochrome, and methemoglobin by its oxidative activity. Due to the high precision of these analyzers, the stringent quality control approaches available, and calibration using commutable reference standards, the measurement of Hb by an automated analyzer is the preferred technique.

In the alkaline hematin method (AHD-575), cetrimide acts by lysing the erythrocytes and precipitating Hb, which conjugates with boronic acid to form a red precipitate that is read at 540 nm.^40^

The WHO hemoglobin color scale is used for estimating Hb concentration from a drop of blood by means of a color scale. The color scale comprises a small card with six shades of red that represent Hb levels at 40, 60, 80, 100, 120, and 140 g/L, respectively (https://www.who.int/medical_devices/publications/en/HbCS_brochure.pdf). This method is more precise than the clinical examination of conjunctiva palm and nail bed and is low cost,^41,42^ but the method should be used only when more precise and accurate devices are not available. Variants of the color scale methods include the Lovibond–Drabkin, Tallqvist, and Sahli’s methods.

The Lovibond–Drabkin technique measures cyanmethemoglobin, and the color of blood is matched with a color standard on discs.^43^

The Tallqvist method is done by placing a drop of blood on a strip of blotting paper and the Hb concentration is interpreted by comparing to color standards on paper.^37^

In Sahli’s method, hydrochloric acid converts Hb to acid hematin, which is then diluted until the color of the solution matches that of the comparator block. This method requires 20 μL of blood, and results can be read 3 min after adding the blood sample to hydrochloric acid. It does not require electricity and is inexpensive.^44^

The copper sulfate method is based on the specific gravity of blood. A blood droplet is allowed to fall into a copper sulfate solution of a specific gravity equivalent to that of blood with known Hb concentration. The determination of Hb concentration is based on the estimation of specific gravity from a blood sample in comparison to a copper solution with a specific gravity value of 1.0532, which corresponds to an Hb concentration of 125 g/L. This method has been used in the past for screening blood donors for anemia.

Currently, most nonspectrophotometric methods are rarely used.^37^

With the availability of new technologies to detect the spectral pattern and concentration of Hb, noninvasive methods are becoming available, although the overall performance is still being examined.^45,46^ Some noninvasive devices use pulse oximetry, while others rely on white light and the capturing of transmission data to measure Hb concentrations in tissue capillaries. These new methods are in the experimental phase and their use in both clinical and population settings requires further research and validation.

Noninvasive transcutaneous pulse co-oximetry has been developed to measure total Hb and its components (oxy-, carboxy-, and methemoglobin moieties) with one manufacturer, Masimo Corp, developing both continuous (Radical-7^™^) and intermittent (Pronto-7^™^) devices. The two devices use different algorithms and the Radical-7 also provides estimates of carboxy- and methemoglobin moieties.

Occlusion spectroscopy is a noninvasive measurement technology featuring a ring-shaped sensor that is attached to the subject’s finger. The sensor temporarily ceases blood flow, initiating an optical signal which yields a high signal-to-noise ratio to estimate Hb concentration.

### Equipment for Hb determinations

Automated Hb analyzers are commonly used for studying blood content and shapes as well as hematocrit and Hb levels. These devices offer higher accuracy and precision at a fraction of the time when compared with manual methods.^11^ Automated hematology analyzers can also measure the size and number of RBCs as well as identify and quantify other blood cells (white blood cells and platelets), resulting in a complete blood count or CBC, which is helpful for diagnosing etiological factors of anemia as well as other diseases or genetic consequences. The initial cost, maintenance, and the laboratory personnel required to use the equipment are high, limiting its extended use, especially in field studies.

In field studies, emergency situations, or settings where resources are limited, portable analyzers, also referred to as point-of-care (POC) devices, such as hemoglobinometers or field photometers, are routinely used. The POC devices are portable, easy to use, and relatively inexpensive, requiring only a small sample of capillary or venous blood and do not require access to refrigeration or electricity and digitally display the Hb value immediately.

In field settings, the HemoCue^®^ device has been used extensively. The HemoCue device provides an immediate numerical Hb value using any type of blood (venous, capillary, or arterial). The blood sample is loaded into a cuvette and undergoes chemical conversion to azide-Hb, with the concentration then measured by absorption photometry at two wavelengths (570 and 880 nm). These were first released in the mid-1980s, and the technology (Hb 201+ system) improved in 2002. The HemoCue 301 and 801 systems, developed after 2008, detect Hb in the same blood samples without this chemical reaction.

The HemoCue 301 quantifies absorbance of oxygenated and deoxygenated Hb, while turbidity is measured and compensated for at 880 nm. Hb concentration is estimated by measuring the absorbance of whole blood at the Hb/HbO2 isosbestic points at the wavelengths of 506 and 880 nm for compensation of turbidity. It requires 10 μL of blood and provides results within 3 s. It can operate in temperatures ranging from 10 to 40°C and measures Hb from 0 to 25.6 g/dL. The HemoCue Hb 801 was released more recently, also requires 10 μL of blood, and results are obtained in less than 1 s. It is used for rapid diagnosis, especially at the patient bedside (https://www.hemocue.us/wp-content/uploads/2021/02/MMUS-01244-Hb-301-Product-Profile.pdf).

The precision (95% CI width is 5 g/L) of these POC analyzers using venous blood from the same individuals is slightly wider than automated analyzers (95% CI width is <3 g/L), which may be due to the use of a smaller blood volume (10 μL against 200 μL) as well as the errors in loading the microcuvettes. However, the variation increases (95% CI width is 12 g/L or more) when using drops of capillary blood collected through finger pricking, even by well-trained and experienced personnel.^47^ This effect has been reported by other researchers.^48,49^ Moreover, a systematic bias for each apparatus, up to 4 g/L, independent of the model, has also been found (Dora Ines Mazariegos, personal communication). The combination of systematic bias of the apparatus with random errors for the use of drops of capillary blood might introduce important errors in the Hb determination of both individuals and populations. The USAID project USAID-Advancing Nutrition is sponsoring an intercountry study^50^ to determine the most appropriate procedures to improve Hb determination using field photometers.

There has been extensive research on HemoCue performance. Studies continuously report higher Hb results with Hemocue Hb-301 compared to the Hb-201+,^51^ and when comparing capillary samples tested by HemoCue with venous samples tested by automated hematology analyzer or cyanmethemoglobin reference methods.^38,52-54^ Several factors may explain the discrepancy between studies, including measurement errors, blood sampling site (capillary vs. venous blood), analytical setting (laboratory vs. field), and population characteristics (healthy adults, children, pregnant women, ill patients).

To diagnose anemia in pregnant women, WHO recommends using an automated hematology analyzer and hemoglobinometer reading in settings where a full blood count is not available.^54^ Screening by hemoglobinometer is recommended over the use of the hemoglobin color scale because research suggests that the hemoglobin color scale is less effective at detecting severe anemia among pregnant women, and the consequences of missing severe anemia are more serious than those of missing mild or moderate anemia.^33,55^

Another reagent-less device is DiaSpect, which measures Hb without a reagent and is based on broad-spectrum photometry. The DiaSpect technology flashes a white light-emitting diode (LED) light through a sample to an optical sensor component. This sensor element identifies the absorbance of the blood at a broad wavelength range, which will provide insight into the overall absorbance spectrum, resulting in a higher specificity and a lower sensitivity to interference (www.ekfdiagnostics.com).

A study from Young et al. in 2021^46^ evaluated the performance of noninvasive devices (smartphone applications and Masimo Pronto) and POC analyzers that use blood samples (HemoCue Hb-301 and Hb-801) against a gold-standard hematology analyzer using venous blood. Noninvasive Hb devices were not well correlated with reference Hb, while HemoCue showed a stronger correlation. The authors conclude that the diagnostic ability of HemoCue was comparable to reference Hb, while noninvasive devices had high user acceptability but considerable biases.

There is a need to identify reliable, reproducible, and comparable equipment for rapid and inexpensive Hb quantification under field conditions and in clinical practice, especially considering including capabilities for the simultaneous detection of markers of inflammation, malaria, or iron or other nutritional deficiencies.

### Preanalytical or sample-related aspects of Hb measurement

Hb can be measured in venous, arterial, or capillary blood. In controlled settings, such as a hospital or clinical laboratory, Hb concentration is generally assessed by automated hematology analyzers, which are very reliable and accurate but expensive and not usually transportable to the field. In general, the measurement of venous blood in a clinical laboratory using the methemoglobin method, either manually or by means of an automated analyzer, is the most frequently used method for estimating Hb concentration and diagnosing anemia.^29^

The situation is different in field/remote settings. Many factors can influence population-based Hb determinations and anemia estimates in population surveys, which in most instances are carried out in environments with limited laboratory capabilities.^9^ Moreover, environmental factors can also affect the reliability of the measurements, such as extreme temperatures or high humidity, geographical inaccessibility of the setting, logistic, and transportation of the samples obtained (e.g., to maintain the cold chain during transportation).

Other factors that could affect the collection of samples, regardless of clinical or field settings, are the social/cultural beliefs of the population regarding blood draw.

An analysis of 11 studies, including children, men, nonpregnant women, and pregnant women from seven countries (Cambodia, India, The Gambia, Ghana, Laos, Rwanda, and United States) exploring factors, such as the blood sampling site (capillary vs. venous), the equipment (HemoCue vs. automated hematology analyzer), and the model of the HemoCue device (201+ vs. 301) that may impact Hb measurements in capillary and venous blood, showed that there was a large variability in Hb concentration measured on capillary or venous blood and using HemoCue Hb 201+ or Hb 301 or automated hematology analyzer.^56^

Substantial bias and considerable imprecision have been reported when comparing capillary and venous sampling for the measurement of Hb. This creates the need for establishing simple and harmonized methodologies for field-based population surveys, which typically use capillary samples to measure Hb and determine the prevalence of anemia,^57^ or avoiding capillary samples and using the preferred blood source, venipunture. The use of capillary or venous blood samples for Hb determinations has been an issue since portable POC apparatuses were developed and proposed to facilitate field work during nutrition and health surveys. Capillary Hb concentrations were significantly greater than venous Hb values when using the same detection apparatus (HemoCue). Also, capillary Hb concentrations were significantly greater than venous Hb values when comparing results from HemoCue versus automatic hematology analyzers.^58^

A study comparing Hb distributions between population-based surveys matched by country and time analyzed four pairs of nationally representative surveys measuring Hb using the HemoCue with capillary (DHS) or venous (BRINDA) blood, matched by country and time. Data included 17,719 children (6–59 months old) and 21,594 nonpregnant women (15–49 years old). Surveys from three of the four countries showed substantial differences in anemia estimates (median: 16 percentage points; range: 1–31) that were consistently lower in BRINDA compared to DHS (2–31 points for children; 1–16 points for women).^59^

The ongoing USAID Advancing Nutrition project mentioned above aims to shed light on the identification of the best procedures and methods for determining Hb concentration in population surveys. Three HemoCue device systems (201+, 301, and 801) are being compared against a certified automatic analyzer when measuring Hb concentration using venous blood from women of reproductive age and children under 5 years of age. Also, it will cover the analysis of performance of the three models when measuring Hb concentration using pooled capillary or capillary blood drop (third drop) against venous blood.^50^

### Quality control of Hb determinations

Quality control at the preanalytical, analytical, and postanalytical phases should be carefully established. Some of the points to address include patient characteristics/disposition/preparation, personnel training, use of international calibration standards, method and frequency of equipment calibration or validation of calibration, blood collection technique, monitoring data collection, recording results, cleaning, adjusting Hb and analyzing results, reproducibility of results, and regular laboratory quality control as per international standards.^9,54,60^

In conclusion, the best practice for Hb determination is the use of venous blood, analyzed on automated hematology analyzers, with high-quality control measures in place. These practices should be reinforced, although in practice and due to logistic reasons, they would probably result in a smaller sample size than the current practice for population surveys.

When these practices are not feasible, the measurement of blood samples using an approved POC device that has been calibrated using standards commutable to the international reference standard could be considered, although considerations about reduction of the sample size might still be needed if venous blood is used. The use of capillary blood is not advised, although the use of pooled drops of blood from a single capillary blood draw might minimize variability in the results and produce results more closely consistent with those using venous blood. More detailed methodologies for appropriate collection of pools of capillary blood are still needed.

In all cases, the blood source, method of sample collection, and method used for Hb determination should be included in any report of anemia at the individual level or when reporting prevalence at the population level; furthermore, it should include a description of the quality control and assurance procedures. Ultimately, data obtained using different blood sources and/or methods would not necessarily be comparable at individual or population levels.

## DIAGNOSING THE UNDERLYING CAUSES OF ANEMIA

### Diagnosing iron-related causes

While essential to diagnose anemia, Hb measurement cannot alone determine the cause. For example, when considering iron deficiency as the cause of anemia, Hb concentration alone is not a suitable indicator for assessing iron status or diagnosing iron-deficiency anemia. For this reason, additional measurements of iron status are needed, such as serum ferritin or serum soluble transferrin receptor (sTfR), which are the most commonly used indicators. Iron status can also be assessed through measurements of total iron-binding capacity, transferrin saturation, zinc protoporphyrin concentration, reticulocyte Hb, erythrocyte protoporphyrin concentration, or in limited cases, bone marrow biopsy.^34^ The use of most of these biomarkers at the population level or in field studies is not always feasible due to cost, equipment, maintenance, training, and the laboratory personnel required.

Ferritin concentration decreases in absolute iron deficiency, but it increases in response to infections and inflammation, and, therefore, its value to interpret absolute iron deficiency under these conditions is limited. Identification of the presence of infection or inflammation for the appropriate interpretation of ferritin values requires the determination of acute-phase proteins. C-reactive protein (CRP) and alpha-1 acid glycoprotein (AGP) are commonly used to assess the concurrent presence of inflammation but are elevated for different durations compared with ferritin. The revised cutoffs for ferritin concentrations used to define iron deficiency at individual and public health levels with proposed adjustments for infection/inflammation were published by WHO in 2020.^61^ For monitoring the effect of interventions on iron status at the population level, WHO recommends using serum ferritin in combination with Hb,^62^ as well as indicators of inflammation.^61^

Serum sTfR concentration is a semiquantitative measure of the extent of the iron deficit in uncomplicated iron deficiency and is less affected by inflammation than ferritin, but concentrations are increased in the presence of accelerated erythropoiesis resulting from hemolysis or ineffective erythropoiesis. The serum transferrin receptor to ferritin concentration ratio (sTfR:SF) can be used to estimate iron status in individuals with iron deficiency, normal iron balance, and increased iron stores.^63^

In 2011, Lynch^64^ described other iron indicators and the clinical information required to determine the causality of iron deficiency in anemia. Serum iron, transferrin saturation, and RBC zinc protoporphyrin (RBC ZPP) would reflect the adequacy of the iron supply for producing RBCs. Serum iron and transferrin saturation are decreased, and RBC ZPP is increased in iron deficiency, although the latter also occurs in inflammatory disorders and due to exposure to lead.

In clinical practice, it is also common to perform a CBC in an automated hematological analyzer, which, in addition to Hb and hematocrit, measures the RBC indices (i.e., mean corpuscular volume, MCV; mean corpuscular hemoglobin, MCH; and mean corpuscular hemoglobin concentration, MCHC), as well as the total and differential counts of white blood cells, platelets, and reticulocyte index. The CBC is also useful to diagnose the causes of anemia, infections, and certain cancers. The reticulocyte count estimates the RBC output from bone marrow and a blood smear helps to evaluate RBC morphology. Other tests that could be requested include a bone marrow biopsy, Hb electrophoresis for Hb variants (i.e., HbS), or other beta globin gene disorders (such as thalassaemia), serum creatinine, erythropoietin, liver function, coagulation profile, or hemolysis profile.^65,66^

The MCV, MCH, and MCHC are generally reduced and RBC distribution width increased in iron deficiency anemia. The reticulocyte Hb concentration is a measure of iron availability to RBCs recently released from the bone marrow and is widely available on modern automated analyzers; it is reduced in iron deficiency.^15^

The use of combinations of indicators may be required to improve the accuracy of estimates of the prevalence of iron deficiency in population samples, although the cost of many of them could limit their practical use.^64,67^

Measurement of hepcidin concentration is emerging as a test for distinguishing absolute from functional iron deficiency. Low hepcidin concentrations indicate a physiological iron need, predict responsiveness to iron, and can enable personalization of the route of iron replenishment.^32^ Both mass spectrometry and immunochemistry-based measurement procedures have been developed to quantify hepcidin concentrations. However, hepcidin levels in the same clinical sample may vary up to a factor of 9 among methods. Effective use of hepcidin measurement in patient care and clinical research require both comparability and analytical reliability to establish uniform clinical decision limits and reference ranges. The use of calibration materials commutable with human plasma or serum will allow standardization that is essential to enable routine clinical hepcidin testing. The recent development of a two-level secondary reference material for hepcidin assays allows worldwide standardization.^68^

### Diagnosing infectious causes

Clinically, anemia of inflammation is diagnosed in patients with normocytic and normochromic anemia (normal MCV and normal MCHC, respectively) in whom there is evidence of systemic inflammation (increased erythrocyte sedimentation rate or CRP level) and evidence of iron restriction that is not caused by systemic iron deficiency (low transferrin saturation along with a high serum ferritin level). The main challenge in establishing a specific diagnosis is the common coexistence of absolute iron deficiency and anemia of inflammation (especially in patients with blood loss from underlying disease) or an iron deficit caused by malnutrition, long-standing inflammation, or increased iron requirements (in growing children, especially during rapid growth spurts, or pregnant women).^69,70^ Due to the complexity of iron metabolism and its effect on various biomarkers, iron deficiency is commonly misdiagnosed. The usual error is a misinterpretation of the laboratory features of the anemia of chronic disease. The serum iron is low, but the iron binding capacity is normal, and ferritin is normal or high. There are problems and exceptions involved in the interpretation of iron indices.

Malaria is one of the primary causes of anemia globally. In 2020, an estimated 241 million cases of malaria occurred worldwide, most of them in the WHO African Region (228 million or 95%).^71^ Iron deficiency is very common in malaria-endemic areas and the relations between iron deficiency and malaria are complex.^72-74^ WHO currently recommends that the provision of iron supplements should be done in conjunction with public health measures to prevent, diagnose, and treat malaria.^75^

Malaria is diagnosed using different techniques, such as microscopic examination of stained thin or thick blood smears or a positive rapid diagnostic test, or molecular diagnostic methods, such as polymerase chain reaction. Other methods being developed include loop-mediated isothermal amplification, nucleic acid sequence-based amplification, isothermal thermophilic helicase-dependent amplification, saliva-based test for nucleic acid amplification, saliva-based test for Plasmodium protein detection, urine malaria test, and transdermal hemozoin detection.^76^

Rapid diagnostic tests are considered highly feasible because they are easy to use, fast, and inexpensive; those detecting the histidine-rich protein 2 (HRP2) may remain positive for weeks because of antigen persistence. The recent emergence and spread of *pfhrp2/3* gene deletions generates a risk of false-negative HRP2 rapid diagnostic test results.^76^ The development of POC devices that simultaneously diagnose malaria and anemia (and, ideally iron deficiency) is of great interest for population studies.

### Diagnosing genetic causes

A highly diverse genetic variation influences Hb concentrations and the risk for anemia. The variants with the largest effects and high frequency are within the *HBA1, HBA2,* and *HBB* genes and are major causes for anemia around the world.^77^ Inherited genetic Hb disorders, particularly the thalassaemia trait and possibly the sickle cell trait, are one of the top three causes of anemia globally. Roughly, 5% of the global population is estimated to carry a significant Hb variant; the percentage is highest in Africa (18%) and Asia (7%). The geographical distribution of this group of diseases is changing and the screening of hemoglobinopathies of high frequency and impact on health is relevant in countries where incidence is increasing. Each year approximately 330,000 children, more than 80% from low- and middle-income countries, are born with a serious inherited Hb disorder, mainly with sickle cell anemia (83%) and a form of thalassemia (17%).^10,78,79^

Diagnosing thalassemia requires a CBC, reticulocyte count, and an Hb electrophoresis or related method for beta thalassemia or sickle cell disease, or genetic testing for alpha thalassemia. For diagnosing sickle cell anemia or to identify people with the sickle cell trait, it is necessary to determine the presence and relative amount of Hb S (which could be done by Hb electrophoresis) in a blood sample or detect mutations in Hb genes. There are POC analyzers that simultaneously diagnose anemia and sickle cell disease/trait.^80,81^

Glucose-6-phosphate dehydrogenase deficiency is an X-linked form of enzyme deficiency affecting more than 400 million people worldwide; it is common in malaria-endemic areas. Although the prevalence is high, most people remain clinically asymptomatic (glucose-6-phosphate dehydrogenase deficiency—NORD [National Organization for Rare Disorders] [rarediseases.org]), but in some cases, it can cause neonatal jaundice, chronic congenital hemolytic anemia, and acute hemolytic anemia. The latter can be triggered by oxidative damage in RBCs due to the reduced activity of the G6PD enzyme: eating fava beans (“favism”), specific medicines, and infections. Diagnosis of this condition is most typically performed using phenotypic tests that measure G6PD enzyme activity in the blood. The deficiency could be suspected in males from Africa, Asia, the Middle East, and the Mediterranean, with a family history of G6PD deficiency and/or by the sudden appearance of intravascular hemolysis with characteristic morphologic changes in the RBCs (e.g., “bite” or “blister” cells).

In summary, at the individual level, after diagnosing anemia by Hb concentration, the abovementioned hematological indices could help understand the causes of anemia and the performance of other hematologic indices. The diagnosis of the main cause of anemia that could guide treatment should contain other tests to be ordered according to the reason for the consult/hospitalization and the clinical signs of the patient to rule out, for example, renal/hepatic disease, cancer, parasitic infections, HIV, tuberculosis, malaria, malabsorptive conditions, micronutrient deficiencies, and/or genetic disorders.

From a population point of view, determining socioeconomic conditions, the prevalence of parasitic infections (including malaria prevalence), genetic Hb variants that affect Hb concentrations, nutritional status of the population, wasting and stunting prevalence, infant mortality, food consumption patterns, environmental pollution (indoor cooking and lead exposure in particular), and water and sanitation conditions could help identify the origin of anemia. From a public health perspective, the diagnosis and burden of iron deficiency, malaria, and genetic variants that affect Hb concentrations are conditions important to consider when addressing the underlying causes of anemia. Table 4 presents some of the basic indicators needed to make the differential diagnostics of anemia.

## MAJOR GAPS AND CHALLENGES FOR ANEMIA DIAGNOSIS

Some of the existing methods for measuring Hb and detecting the common causes of anemia are expensive or too complex for use in primary health care settings in resource-limited countries where anemia is most prevalent or for population-based survey assessments determining prevalence.

Assessment of anemia is often difficult in low-resource settings due to a lack of or poor maintenance/calibration of equipment, limited access to reagents or trained laboratory personnel, unreliable electricity, as well as logistics and maintaining the cold chain for population-based surveys, among other factors.

More accurate and accessible field-based diagnostic and POC screening, diagnostic, and monitoring tools are needed, especially for settings where there is no access to a well-equipped laboratory. Factors such as accuracy, precision, cost, availability of reagents, and the need for electricity or fulfilling of the minimal operating conditions required by the test should be considered for the various settings and use cases (e.g., population-based surveys, community surveys, peripheral health facilities, and referral hospitals).

Especially in field-based conditions, there is an urgent need for simple but reliable devices for the *simultaneous,* inexpensive assessment of Hb, malaria, parasitic infections, ferritin, CRP/AGP, and/or hepcidin, especially those that could provide a combination of biomarkers to obtain a diagnosis of the cause of anemia, such as the simultaneous detection/quantification of anemia, malaria, and inflammation-adjusted iron deficiency.

Hb cutoffs to define anemia are being reviewed and the inclusion of other age groups (6–24 months, 24–59 months) and adjustments by altitude or smoking are being reviewed. Currently, there is no agreement on a cutoff for diagnosing anemia in infants under 6 months of age. Also, there is a single Hb cutoff for children from 6 to 59 months of age, this being a population most vulnerable to the detrimental impact of anemia, with large physiological differences during the first years of life, suggesting that risk might vary among this group and benefit from further granularity by examining thresholds among narrower age groupings.

Results from clinical and field studies indicate that there are differences in Hb content in venous and capillary blood samples.^9,57,59^ Capillary Hb concentrations estimated using drops of blood collected through finger/heel pricking were significantly greater than venous Hb values between POC equipment and when compared to automatic hematology analyzers. The adoption of good clinical practices and the preferable use of venous blood, as well as periodic calibration of equipment, should be encouraged to reduce errors. The use of pooled capillary drops instead of individual drops warrants further study.

In clinical settings and for individual care, the use of venous samples and the examination of hematological indexes along with other tests according to the reason for the consult/hospitalization and the clinical signs of the patient should be encouraged since these measures could help to support the accurate determination of anemia, identify the main causes of anemia, and guide treatment.

Diagnosis of genetic Hb disorders (thalassemia, glucose-6-phosphate dehydrogenase deficiency, and sickle cell trait) could be performed by a blood test. The feasibility for a rapid test combining the detection of those disorders and Hb concentration could be investigated. There have been important efforts for inexpensive, rapid tests for sickle cell diagnosis (https://maternova.net/products/sickle-cell-test; https://www.sysmex-europe.com/products/products-detail/hemotypesc.html; https://www.biomedomics.com/products/hematology/sicklescan/), and also for glucose-6-phosphate dehydrogenase deficiency.^82^

## CONCLUDING REMARKS

Anemia should be considered as an outcome of nutritional, physiological, social, and environmental determinants. The diagnosis of anemia and the identification of its cause(s) are required to address and tackle anemia and the subjacent cause(s).

The best practice for Hb determination is the use of venous blood, analyzed on automated hematology analyzers, with high-quality control measures in place. The importance of a correct anemia diagnosis is highlighted by the cost of a misdiagnosis. A false-negative diagnosis may result in missing out and not treating anemia, its causes, and its adverse effects. On the other hand, a false-positive diagnosis may result in the provision of unneeded treatment and risk of excess iron therapy, or referral for expensive laboratory tests to determine a cause of anemia, wasting valuable resources and risking producing harm.

At the individual level, clinicians must understand the causes of absolute and functional anemia to diagnose and treat anemia at the clinical level. Actions toward anemia diagnosis and control at public health levels will require global, regional, and country actions, that should cover general and context-specific characteristics.

Research into the biology, epidemiology, diagnosis, and treatment of anemia could result in new tools to diagnose anemia and its determinants in a single, more affordable, field-friendly, and less time-consuming test. The development of the sandwich ELISA and other similar products to measure multiple indicators concurrently has facilitated the determination of risk factors of anemia, although commutability studies are pending.^60^ For other indicators to assess causes of anemia, relatively large volumes of blood are needed, the costs are higher, and specialized laboratories are required.

Establishing a correct diagnosis of anemia and its causes would also support the WHO *Comprehensive framework for integrated action on the prevention, diagnosis and management of anemia* that seeks to provide strategic, effective, and implementable actions to reduce anemia, accelerate progress toward the global target on anemia, and optimize health and well-being. Stakeholders are required to understand and be convinced that anemia prevention and management require a multisectoral and coordinated response.

## Figures and Tables

**TABLE 1:** Classification of public health significance of anemia in populations on the basis of prevalence estimated from blood levels of hemoglobin.^29^

Category of public health significance	Prevalence of anemia (%)
Severe	40 or higher
Moderate	20.0–39.9
Mild	5.0–19.9
Normal	4.9 or lower

**TABLE 2:** Hemoglobin levels (g/L) to diagnose anemia at sea level.^29^

Population, age	No anemia	Anemia		
		Mild	Moderate	Severe
Children, 6–59 months	≥110	100–109	70–99	<70
Children, 5–11 years	≥115	110–114	80–109	<80
Children, 12–14 years	≥120	110–119	80–109	<80
Nonpregnant women, 15 years and above	≥120	110–119	80–109	<80
Pregnant women	≥110	100–109	70–99	<70
Men, 15 years of age and above	≥130	110–129	80–109	<80

**TABLE 3:** Summary of various method and analyzer characteristics used for hemoglobin measurement

Method/analyzer	Setting	Method principle	Blood volume (μL)	Cost/test CM as reference^a^	Calibration reagents
Cyanmethemoglobin method (CM)^a^	Clinical laboratory	Hb is converted into methemoglobin, and to cyanmethemoglobin by adding potassium cyanide and ferricyanide. Absorbance is then measured at 540 nm using a photoelectric colorimeter against a standard quality control solution.	10–200	1 US$	Drabkin’s reagent
Automated hematology analyzers	Clinical laboratory	Hb is converted into methemoglobin, cyanmethemoglobin, or other Hb derivatives, and measured at the wavelength that is more specific for the Hb derivative. They can also identify and measure physical characteristics of the red blood cells and other blood cells.	200	5–10× CM	Quality control material specific to the analyzer
Point-of-care photometric analyzers (e.g., HemoCue, Hemo-Control, Hb-Quick, DiaSpect, URIT, and TrueHb)	Clinical laboratory and field settings	For the Hb-201+, Hemo-Control, and Hb-Quick, Hb is converted to methemoglobin by sodium nitrate from the ferrous to ferric state to form azide-methemoglobin, where the Hb concentration is then detected at 570 and 880 nm and read using a photoelectric colorimeter. For the Hb-301, the Hb concentration is simply determined by the photoelectric colorimeter.	10	2–4× CM	Not required for Hb-201+, Hb-301, Hemo-Control, DiaSpect, TrueHb, and URIT, but HemoTrol and EuroTrol have liquid controls
Paper- or color-based analytical devices (e.g., μPADs and color-based filter test)	Clinical laboratory chromatography paper with a wax finish that is heated at 150°C for 3 min	Blood samples are diluted with Drabkin’s reagent and then incubated for 10 min. A 20 μL sample of blood is then placed onto the paper-based analytical device, and the Hb concentration is then read using a portable flatbed scanner after the sample dries.	20	2× CM	Drabkin’s reagent
WHO color scale	Clinical laboratory and field settings	Contains six shades of red (i.e., lighter to darker, corresponding to an Hb concentration of 40, 60, 80, 100, 120, and 140 g/L) that are mounted onto strips. A drop of blood is placed onto a moveable piece of filter and compared to the shades of red on the color scale.	30	<CM	None
Color-based analytical devices	Clinical laboratory and field settings	Uses small round tube with a cap that holds the solution, which mixes with the blood sample that enters the device via capillary action. The sample is then compared to a color chart with a range of colors.	5	=CM	Drabkin’s reagent
Sahli method	Field settings	Hydrochloric acid converts hemoglobin to acid hematin, which is then diluted until the color of the solution matches that of the comparator block.	20	<CM	Hydrochloric acid
Copper sulfate method	Clinical laboratory	Hb concentration based on the estimation of specific gravity from a blood sample. Specific gravity value of 1.053 corresponds to an Hb concentration of 125 g/L.	10	=CM	Fresh anticoagulated blood samples
Noninvasive analyzers (e.g., Masimo)^b^	Clinical laboratory	CO-oximeter measures the oxygen saturation, pulse rate, perfusion index, and total Hb by detecting the levels of oxygen and carbon monoxide bound to Hb in the individual. This is done by placing a probe on the finger.	–	2–5× CM	–

*Note:* Adapted from Ref. 9.

Abbreviation: Hb, hemoglobin.

a Cyanmethemoglobin method is used as the reference for cost comparisons.

b Does not require a blood sample.

**TABLE 4:** Some of the basic indicators to be considered for the differential diagnosis of anemia

Etiology of anemia	Indicators to address iron metabolism	Determinants	Other indicators to identify cause
Iron and other nutritional deficiencies	Hemoglobin concentration + Ferritin Serum iron TIBC sTfR sTfR:SF Tf saturation Hepcidin RBC ZPP CHr CBC CRP/AGP Blood smear Bone marrow aspiration (rarely)	Iron, copper, zinc, vitamins A, B2, B6, B9, B12, C, D, and E deficiencies Dietary habits Increased loss of blood Increased requirements	Serum/plasma/urine/hair concentrations of the suspected micronutrient/vitamin deficiency 24-h food consumption recall questionnaire Menstrual losses Anthropometric status/nutritional status Cooking practices that might influence micronutrient absorption Comprehensive questionnaire on dietary habits, contraception, blood losses (menstrual, gastrointestinal), birth spacing, education/social level, genetic background
Infection and inflammation	Hemoglobin concentration + Ferritin Serum iron TIBC sTfR sTfR:SF Tf saturation Hepcidin RBC ZPP CHr CBC CRP/AGP Blood smear Bone marrow aspiration	Malaria, tuberculosis, soil-transmitted helminth infections, schistosomiasis, HIV, *Helicobacter pylori* infection, cancer, low-grade inflammation	Serum cytokines and inflammation markers (CRP/AGP) Microbiological detection of infection (bacteria/virus/parasite, chronic conditions) Malaria microscopic examination of stained thin or thick blood smears or rapid diagnostic tests, or molecular diagnostic methods (PCR) Stool parasitic count for soil-transmitted helminth infections *Schistosoma* eggs in feces and urine Blood test also possible Liver function, coagulation profile, hemolysis profile, serum creatinine Comprehensive questionnaire and clinical examination
Genetic red cell disorders	Hemoglobin concentration + Ferritin Serum iron TIBC sTfR sTfR:SF Tf saturation Hepcidin RBC ZPP CHr CRP/AGP Blood smear	Sickle cell anemia Thalassemia Glucose-6-phosphate dehydrogenase deficiency	Complete blood count (CBC) Genotyping for alpha thalassemia, and hemoglobin electrophoresis for beta thalassemia Electrophoresis of hemoglobin for HbS G6PD enzyme activity Clinical history and examination
Social, behavioral, and environmental determinants	Hemoglobin concentration + Ferritin Serum iron TIBC sTfR sTfR:SF Tf saturation Hepcidin RBC ZPP CHr CBC CRP/AGP Blood smear Bone marrow aspiration	Water hygiene and sanitation Economic, political, institutional capacity/resources Climatic/environmental conditions Multiple pregnancies Educational level Religion/cultural beliefs Access to care/services	Access to safe drinking water Data on social, economic, and health statistics Community/women empowerment assessment Access to health facilities Availability of antenatal care Deworming, WASH, family planning, and/ormalaria prevention campaigns Ongoing food fortification, micronutrients supplementation programs Food security Comprehensive questionnaire

Abbreviations: CBC, complete blood count; CHr, reticulocyte hemoglobin content; CRP/AGP, C-reactive protein, alpha-1-acid glycoprotein; RBC ZPP, red blood cells protoporphyrin; sTfR, soluble transferrin receptor; sTfR:SF, soluble transferrin receptor to ferritin ratio; TIBC, total iron binding capacity; WASH, water access, sanitation, and hygiene.
